# Bacteria Detection and Differentiation Using Impedance Flow Cytometry

**DOI:** 10.3390/s18103496

**Published:** 2018-10-17

**Authors:** Casper Hyttel Clausen, Maria Dimaki, Christian Vinther Bertelsen, Gustav Erik Skands, Romen Rodriguez-Trujillo, Joachim Dahl Thomsen, Winnie E. Svendsen

**Affiliations:** 1 SBT Instruments ApS, Diplomvej 381, 2800 Kgs. Lyngby, Denmark; chc@sbtinstruments.com (C.H.C.); cvb@sbtinstruments.com (C.V.B.); ges@sbtinstruments.com (G.E.S.); 2 Technical University of Denmark, Department of Micro- and Nanotechnology, Oersteds Plads 345 East, DK-2800 Kgs. Lyngby, Denmark; jdth@nanotech.dtu.dk (J.D.T.); wisv@nanotech.dtu.dk (W.E.S.); 3 Department of Electronics and Biomedical Engineering, University of Barcelona. C/Martí i Franquès 1, 08028 Barcelona, Spain; romen.rodriguez@ub.edu; 4 Institute for Bioengineering of Catalonia (IBEC), the Barcelona Institute for Science and Technology (BIST), C/Baldiri i Reixac 10-12, 08028 Barcelona, Spain

**Keywords:** electrical impedance spectroscopy, bacteria detection, bacteria differentiation, water quality, bacteria counting

## Abstract

Monitoring of bacteria concentrations is of great importance in drinking water management. Continuous real-time monitoring enables better microbiological control of the water and helps prevent contaminated water from reaching the households. We have developed a microfluidic sensor with the potential to accurately assess bacteria levels in drinking water in real-time. Multi frequency electrical impedance spectroscopy is used to monitor a liquid sample, while it is continuously passed through the sensor. We investigate three aspects of this sensor: First we show that the sensor is able to differentiate *Escherichia coli* (Gram-negative) bacteria from solid particles (polystyrene beads) based on an electrical response in the high frequency phase and individually enumerate the two samples. Next, we demonstrate the sensor’s ability to measure the bacteria concentration by comparing the results to those obtained by the traditional CFU counting method. Last, we show the sensor’s potential to distinguish between different bacteria types by detecting different signatures for *S. aureus* and *E. coli* mixed in the same sample. Our investigations show that the sensor has the potential to be extremely effective at detecting sudden bacterial contaminations found in drinking water, and eventually also identify them.

## 1. Introduction

One of the most common ways to determine if bacteria are present in a given sample, e.g., food, urine, blood etc. is to plate the sample on an agar plate and culture it for 1–3 days inside an incubator. This method is extremely easy and precise, but also very time consuming, because you need to wait for the growth to occur before you know the concentration of your sample and can react accordingly.

This problem is of particular importance in drinking water systems [1,2], because the contaminated water can quickly affect a large population. The slow analysis time of a traditional plate count means that the water utilities cannot alert the population that a contamination is present before the potentially dangerous water is consumed. Due to the size of the piping system, it can also be difficult for the utilities to identify the source and location of the contamination and the drinking water can stay polluted for many days.

Effective enumeration of bacteria in liquid samples without the need for a time consuming culture step has been an issue for several years [3] and a number of methods already exist that address this problem. Some of the most prominent of these are fluorescence flow cytometry [4], electrical impedance spectroscopy [5] and image analysis methods [6]. However, fluorescence flow cytometry requires the use of either stains or labels in order to enumerate bacteria, which complicates the process [7]. Imaging techniques require the development of identification algorithms and are difficult to perform in real time.

Characterizing biological samples by detecting dielectric changes using electrical impedance is a technique that has been used in several forms, including Coulter counters and impedance flow cytometers. Electrical impedance spectroscopy (EIS) [8,9,10,11] has been used to investigate different biological samples in suspension and is able to characterize biological sample properties in a label free manner. In EIS, a microfluidic channel is used to direct a sample of particles dispersed in a liquid towards a set of electrodes with an applied AC electrical field [10]. Changes in the electric field during particle transitions depend on the dielectric and structural properties (size, composition) of the particles, which can therefore be determined through interpretation of the measured electrical current.

Different properties of the particles are probed at different frequencies, e.g., the particle size is probed at low frequencies in the kHz range while the particle composition is probed at higher frequencies in the low MHz range. Therefore, modern EIS devices apply a mixed multi-frequency signal in order to simultaneously probe the particle properties. Integration of EIS into microsystems is a relatively new development, which has been demonstrated to have various applications within characterization of biological samples [8,9]. The advantages of this integration are better control and higher sensitivity of the system.

The technique has been widely used for analysis of biological material; from differentiation of red blood cells extracted from fish and human leukocytes [12] to detection of DNA in droplets [13]. Gawad et al. [8] presented the first single cell EIS differential microfluidic cell analysis system. They reported the capability of EIS to differentiate erythrocytes and erythrocyte ghost cells, as well as solid particle size separation in continuous flow. Furthermore, the technology has been applied to a broad number of different micro-sized samples; it has been used to distinguish between different yeast cells [14] and human blood cells of different kinds [9,15,16,17]. EIS has also been used to measure the effect of electrical lysis on yeast cells [18]. Additionally, several reports exist on the modeling of the signal response and how to elucidate how the different properties of the sample influence the recorded signal [14,19]. Further applications include differentiating bacteria from polystyrene beads [20] and distinguishing heat treated bacteria samples from un-treated samples [11].

**Figure 1:**
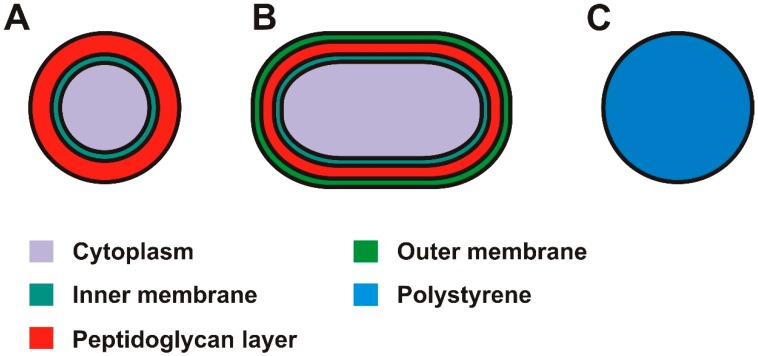
Schematic drawings of the bacteria and particles used in this report (**A**) Gram-positive *S*. *Aureus* (**B**) Gram- negative *E. coli*. (**C**) Polystyrene beads.

Millions of bacteria species exist, many with unique structural and pathogenic properties. Traditionally, bacteria are divided into two classes, gram negative and gram positive, depending on the ability of their cell wall to retain crystal violet dye. Gram positive bacteria have an outer membrane layer (see Figure 1), which is not present for gram negative bacteria. The presence of the extra membrane is therefore a potential target for differentiation between gram positive and gram negative bacteria by EIS.

In this paper we will present how we can detect and enumerate bacteria in water samples using EIS. We will show that it is possible to detect bacteria not only in artificial buffers (e.g., diluted PBS), but also in tap water. Furthermore, we will show that it is possible to differentiate between gram positive and gram negative bacteria, by using our method to distinguish between two of the most commonly found bacteria. The novelty of the paper lies in the real time continuous detection of bacteria in water samples using impedance, as opposed to publications detecting bacteria in a static environment.

## 2. Materials and Methods

### 2.1. Sample Composition

The bacteria used in this work are *Escherichia coli* (*E. coli*) and *Staphylococcus aureus* (*S. aureus*). *E. coli* is a rod shaped Gram negative bacterium with a length of 2–3 µm [21] and a diameter of 0.5 µm. It is a common cause for bacterial infections in humans and animals [22]. The cytoplasm of *E. coli*, which is considered to be electrically conductive, is surrounded by an electrically isolating lipid inner membrane (10 nm [23]), a conductive periplasmic space containing the peptidoglycan wall (20 nm [24]) and another isolating lipid outer membrane (13 nm), see Figure 1B.

*S. aureus* is a spherical Gram positive bacterium with a diameter of approximately 1 µm [25]. It has a conducting cytoplasm in the center surrounded by a lipid membrane and a peptidoglycan cell wall, characteristic of gram-positive bacteria. The lipid membrane has a thickness of 10 nm and is normally assumed to be non-conducting, while the cell wall has a thickness of 60 nm and is assumed to be electrically conducting [24], see Figure 1A.

The bacteria (*Escherichia coli* strain INV-α from Invitrogen, Nærum Denmark, methicillin-susceptible *Staphylococcus aureus* from Statens Serum Institut, Copenhagen, Denmark), were cultured on appropriate pre-prepared agar plates (LB agar, L5542-10EA, Sigma Aldrich Denmark A/S, Copenhagen, Denmark; blood agar, A600, VWR, Copenhagen, Denmark, respectively). The bacteria were spread on the agar plates, dried (10–15 min), and incubated at 37 °C for 24 h. Before the experiments a small amount of *E. coli* was transferred from the agar plate into individual incubation tubes containing 5 mL of LB broth and incubated at 37 °C for 24 h with rotation (215 rpm). After the 24 h incubation 1 mL LB broth containing bacteria was centrifuged, the broth was removed, and the bacteria were re-suspended in either diluted PBS (1/20× PBS, D8537, Sigma Aldrich) or tap water at appropriate concentrations. Polystyrene beads, illustrated in Figure 1C, with diameters of 1 µm and 2 µm were used as non-biological reference particles. The polystyrene beads are dielectric homogenous spheres which conduct current primarily on their surface [26]. The polystyrene beads were acquired from Polysciences, Inc. (Warrington, PA, USA).

### 2.2. Detection Principle

**Figure 2:**
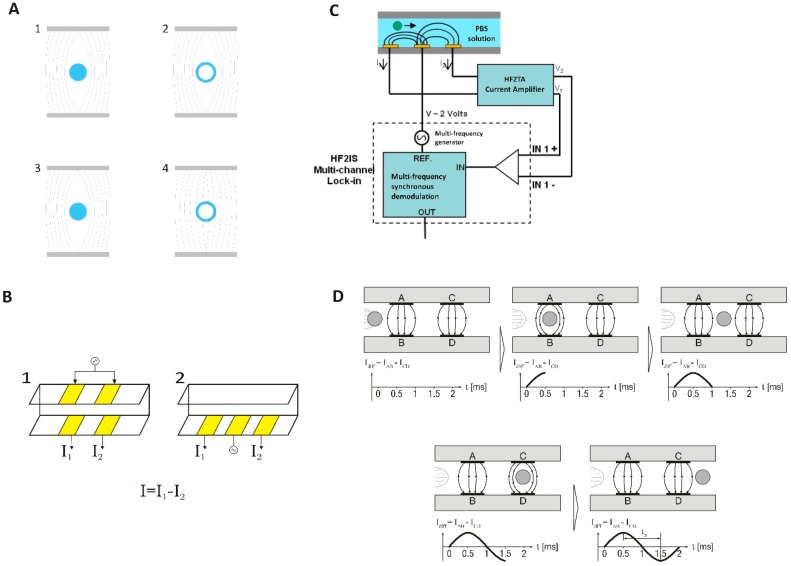
(**A**) Electric fields generated by two electrodes at low frequencies (1 and 2) and high frequencies (3 and 4) on beads (1 and 3) and bacteria (2 and 4). (**B**) Schematic of the measuring principle using front facing (1) or coplanar (2) electrodes. A differential signal between the two measuring electrodes is recorded for further analysis. (**C**) Schematic drawing of the measurement setup, in the case of coplanar electrodes. The sample is injected into the system by a syringe pump. A multi-frequency lock-in amplifier is used to generate and detect the signal. The signal from the measuring electrodes is passed through a current preamplifier before it is returned to the lock-in amplifier. (**D**) Schematic showing the detection principle. When the particle is not influencing the electric field generated by the electrodes, the differential signal is zero. As the particle travels in the channel it will only disturb the field of one set of electrodes at a time, which gives rise to a differential signal. When the particle leaves the electrodes area a transition looking like a sinusoidal signal will have been recorded, indicating the transition of one particle.

**Figure 3:**
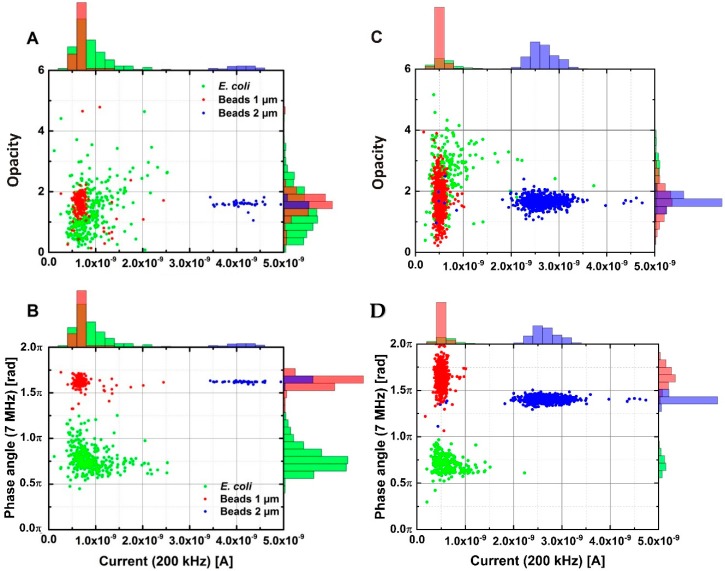
(**A**) Opacity plotted against the low frequency signal of *E. coli* and polystyrene beads in drinking water. (**B**) Phase shift at 7 MHz plotted against the low frequency signal of *E. coli* and polystyrene beads in drinking water. (**C**,**D**) Same as (**A**,**B**), but with the bacteria and beads dispersed in 1/20× PBS.

**Figure 4:**
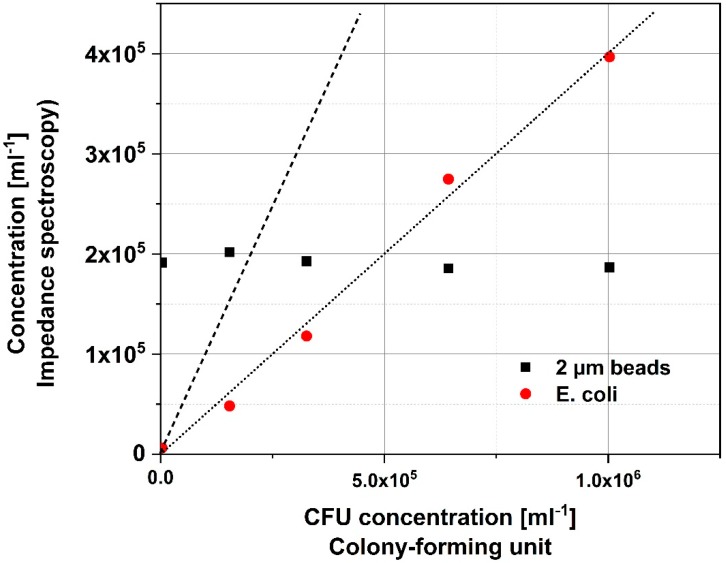
Bacteria concentration measured with the system (impedance spectroscopy) and colony-forming unit counting. The beads were only counted using the system and their concentration was kept constant at 2 × 10^5^ mL^−1^. The dashed line represents the 1:1 relation between the two methods. The dotted trend line for the *E. coli* data has an *R*^2^ value of 0.9937.

Figure 2A illustrates the detection principle as well as the electrode configuration on the microfluidic chip. At low frequencies (100–1000 kHz) (Figure 2A, 1 and 2) it is expected that the electrical field will not be able penetrate the beads or the bacteria due to the isolating nature of their bulk and membrane, respectively. The current will only move in the liquid medium and the measured signal will depend on the volume displacement due to the particles or bacteria investigated, i.e., the signal depends on particle/bacteria size. At higher frequencies (1 MHz–10 MHz), the electric field is still not able to penetrate the bulk of the polystyrene beads (Figure 2A, 3). However, it is capable of partially penetrating the membrane of the bacteria and thus probe the membrane and cytoplasm composition (Figure 2A, 4) [19].

### 2.3. Chip Fabrication

Two types of chips were fabricated and used in this work: chips with coplanar electrodes and chips with front facing electrodes (Figure 2B). The fabrication process is similar for the two chip types: Gold electrodes were defined on 4-inch Pyrex wafers by photolithography, e-beam vapor deposition and lift-off, using titanium as an adhesive layer. On top of the electrodes the channels were formed in SU-8 2005 (MicroChem, Berlin, Germany) by negative photolithography as described by Demierre et al. [27,28]. The channels were sealed using a second Pyrex wafer (lid wafer) with openings for electrode access and fluidic inlet and outlet defined using powder blasting. The lid wafer used for the front facing electrodes had additionally gold electrodes fabricated as those of the bottom wafer. The lid wafer was then thermally bonded to the bottom wafer as described by Serra et al. [29]. The microchannels were 10 μm wide and 10 μm high. The front facing electrodes exposed to the channel are 10 μm long and 10 μm wide with a pitch of 16 µm. The dimensions and pitch are the same for the coplanar electrodes.

### 2.4. Measurement Setup

The setup used in this work uses a layout as shown in Figure 2B. An AC excitation signal is applied to either the top electrodes (Figure 2B 1, front facing) or the middle electrode (Figure 2B 2, coplanar), and the current is measured at the two remaining electrodes. In order to normalize the detected signal a differential measurement is carried out between the two sets of electrodes, giving a measured current of *I* = *I*1 − *I*2, as shown in Figure 2B.

The measurement setup consists of a custom-built aluminum chip holder containing the necessary electrical and fluidic connections (fittings from Upchurch Scientific^®^, IDEX Health and Science, Oak Harbor, WA, USA) for the microfluidic chip. O-rings were used to seal the fluid connections of the chip to the holder. Differential EIS measurements were performed with a HF2IS Impedance Spectroscope (Zurich Instruments, Zurich, Switzerland). The peak differential current during a transition was used as the characterizing parameter of the particle. The signal was amplified by a HF2TA trans-impedance amplifier (Zurich Instruments). A schematic drawing of the setup is shown in Figure 2C. The applied signal was 3 V (amplitude) with the attenuation of the low pass filter of the lock-in set to 24 dB with a bandwidth of 502 Hz. The sample rate was 28,800 Sa/s. The preamplifier trans-impedance gain was set to 10 kV/A.

The sample liquid was driven through the chip using a Nexus 3000 syringe pump (Chemyx Inc., Stafford, TX, USA) at a rate of 0.01 μL/min. The measurements were carried out at two frequencies simultaneously; a low frequency of 200 kHz and a high frequency of 7 MHz. These frequencies were selected based on experimentally recorded spectra at frequencies between 200 kHz and 10 MHz on samples containing different bacteria (*E. coli*, *S. aureus* and *L. anisa*–data shown in Supplementary Figure S1).

### 2.5. Samples

The experiments were generally performed in a low conductivity saline solution (PBS diluted to 1/20 with Milli-Q water) in order to better control the conductivity of the medium and to ensure that the medium was particle free before introducing *E. coli* and polystyrene beads. The conductivity of the prepared saline solution was measured to be 85 mS/m with a CDM210 conductivity meter (Radiometer Analytical, Lyon, France).

Additionally, experiments in tap water were carried out. The samples were prepared in tap water to demonstrate the capabilities of the biosensor in its envisioned operational environment, considering that solid particles often exist in tap water supply and hence should be distinguishable from bacteria contamination.

The concentration of bacteria (*E. coli* and/or *S. aureus*) and beads of 1 and 2 µm diameter in the two sample buffers was always 2.5 × 10^6^ mL^−1^ for each particle type. The beads were used in order to test the system’s ability to discriminate solid particles in the 1 µm to 2 µm range from bacteria (which have approximately the same size) in tap water.

For the experiments investigating the sample concentration, the bacteria (*E. coli*) concentration was varied while the concentration of 2 µm beads was kept constant at 2 × 10^6^ beads/mL.

### 2.6. Data Acquisition and Analysis

The raw data was recorded by a computer and analyzed using a custom MATLAB script (MathWorks Inc., Natick, MA, USA), which identifies the differential peak current (Figure 2D) during a particle transition. The program identifies the height and width of the peaks from the transitions for the signal at both frequencies. The concentration of particles (*C*) is calculated using the transition time (*t_trans_*) (i.e., the time is takes a particle to pass from one measurement electrode to the next) and the volume (*V*) between the electrodes together with the number of events (*N*) during the time of a measurement (*t_m_*):

*C* = (*N*⁄*t_m_*)/(*V*⁄*t_trans_*) = (*N* × *t_trans_*)/(*V* × *t_m_*) (1)

In this way, the calculated concentration is independent of the flowrate of the sample.

## 3. Results and Discussion

### 3.1. Separation of Biological and Non-Biological Samples in Diluted PBS and Tap Water

Samples in tap water containing *E. coli*, 1 μm and 2 μm beads were driven through the chip. Figure 3A,B show a correlation plot of the peak differential current measured at 200 kHz versus the opacity ((high frequency peak differential current)/(low frequency peak differential current)) versus the phase angle response measured at 7 MHz, respectively. We observe a clear separation in the measured current response at 200 kHz for 1 µm and 2 µm beads. *E. coli* and 1 µm beads have roughly the same volume, and thus provide the same current response at the low frequency. As seen in Figure 3A, a plot of the opacity versus current is not able to differentiate between bacteria and beads of the same volume. However, a clear separation between 1 µm beads and bacteria can be seen in the phase angle measurements at 7 MHz. It is also observed that the 1 µm and 2 µm beads share the same phase angle response due to their similar bulk composition. It is reasonable to assume that the membrane structure of *E. coli* introduces a phase response at 7 MHz, which is different from the signal from the polystyrene beads, which do not have a membrane. This makes the phase angle a potentially useful parameter for differentiating microorganisms from other particles in tap water.

Tap water can be a difficult buffer to work with as its composition and electrical properties vary depending on the time of day and the source. In order to have a well-controlled buffer with a conductivity in the same range as tap water we used PBS diluted 20 times with milli-Q water (1/20× PBS), which has a conductivity of 0.085 S/m. *E. coli*, 1 µm and 2 µm beads were added to the buffer and the impedance response was measured with the sensor. The results are shown in Figure 3C,D. It is evident that the two buffers behave similarly, with the correlation plot of the current at 200 kHz versus the phase angle at 7 MHz still showing a clear separation between bacteria and beads. The small differences in the phase of the 1 and 2 µm beads in Figure 3D can most likely be attributed to the function of the filters in the measurement setup and variations in the data analysis algorithm.

Figure 3 confirms that 1/20× PBS is a good substitute to tap water when one wants to control the properties of the liquid better, considering that tap water comes in a variety of conductivities and composition. We note here that the ionic strength (and effectively the conductivity) of the solution has an effect on the recorded high frequency phase. Tap water comes in a range of different conductivities, but it should preferable be between 0 and 150 mS/m to be suitable for human consumption. In this range of conductivities the phase signal from the bacteria is stable, as is shown in Figure S2 (Supplementary Data), and we can therefore conclude that local variations in drinking water conductivity will not have an effect on the sensor. However, we note that at higher conductivities the phase signal will change, e.g., if PBS is used as the solution, in which case the bacteria can no longer be distinguished from polystyrene beads (Figure S3 in Supplementary Data).

### 3.2. Determination of Bacteria Concentration

It is not essential to know the exact bacteria concentration when detecting sudden contamination events, however, it is very important to be able to detect a change in the bacteria concentration, as it is this change in concentration, which is used to determine if the water supply has been suddenly contaminated. As a result, any bacteria detection system should be capable of rapidly determining any increase in bacteria concentration.

To demonstrate the sensor’s ability to accurately measure changes in concentration five samples with varying *E. coli* concentration and a fixed concentration of 2 µm beads were prepared (see Table 1). The concentration was then determined with the sensor with a measurement time of 25–30 min per sample. The samples were also plated and the colonies were counted after 24 h. The calculated concentrations by the EIS system (Equation (1)) and the plate count are shown in Table 1.

The plots shown in Figure 4 depict the measured data of the bacteria concentration as detected by the system and by CFU counting. The bacteria count of the system is not identical to the results obtained with the CFU method, but there is a proportional relation between the measured values by EIS and the values obtained by CFU. This indicates that the difference between the two counting methods is caused by the way the concentration is estimated (see Section 2.6), for example due to variations in the detection volume that arise from fabrication. The result could also be influenced by smaller bacteria, which are missed due to a lack of sensitivity in the system. The 2 µm beads are all counted successfully as the signal from these is at least 8 times larger than that for the bacteria. Even though the system is not able to accurately determine the exact concentration of bacteria in a sample, its ability to precisely determine a change in concentration makes it a candidate for monitoring bacteria contaminations in liquid samples. Moreover, since the relationship between the actual (by CFU) concentration and measured concentration is linear, then the actual concentration can be accurately estimated after a system calibration.

We note that the discrepancy observed in sample A, where the concentration calculated by EIS is larger than the one found by CFU which arises due to statistical variations in low concentration samples along with the in-built errors in the data processing. Indeed, we note that in the 25 min of the experiment only seven transitions were registered as opposed to about 100 and over in samples B to E. As the data analysis software can erroneously detect up to five transition events in a clean sample, it is likely that the EIS calculated concentration for sample A is overestimated. From Table 1 and using samples B to E we can also estimate our detection limit as the concentration required for detecting a single transition event as 522 ± 40 bacteria/mL.

### 3.3. Differentiation between Gram-Positive and Gram-Negative Bacteria

**Figure 5:**
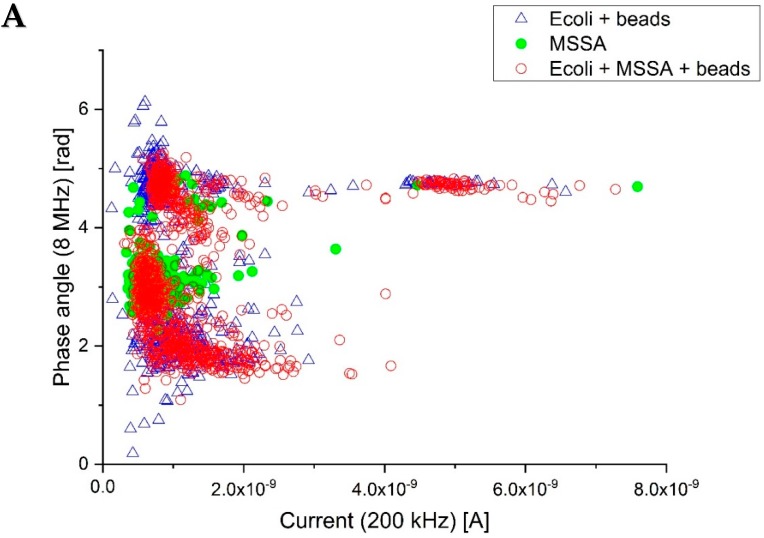

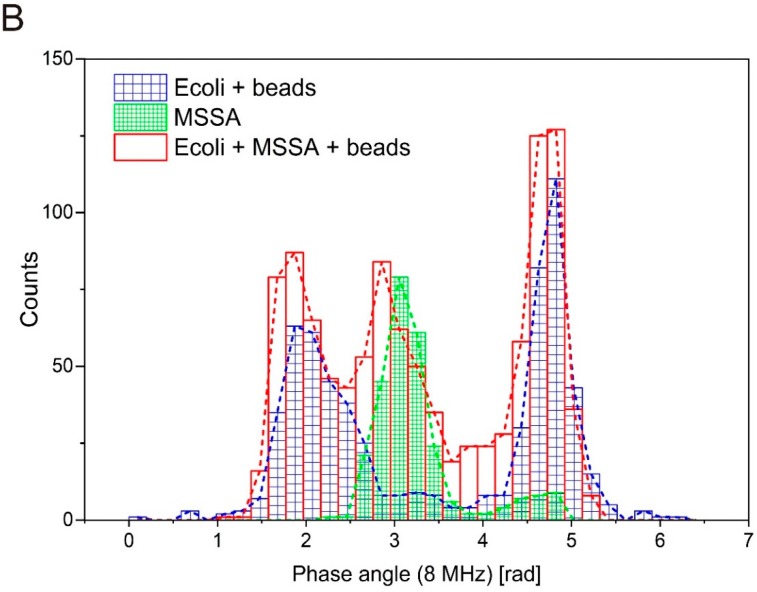
(**A**) Phase shift at 8 MHz plotted against the low frequency signal of the bacteria *E. coli* and *S. aureus* and polystyrene beads in diluted PBS (**B**) Histogram of the high frequency phase angle, clearly showing the two populations of bacteria.

The structural differences between the membranes of Gram-positive and Gram-negative bacteria make it probable that a difference will appear on the high frequency phase signal. This is indeed the case, as can be shown in Figure 5. Figure 5A shows the correlation plot for three samples, one containing *E. coli* mixed with polystyrene beads, one containing *S. aureus* (MSSA) and one containing a mixed population of *E. coli*, MSSA and beads. In Figure 5A we can see that distinct populations appear around 2, 3 and 5 rad, with the population at 5 rad visible in all the samples, suggesting that this population comes from the polystyrene beads that were present in two of the samples. As the MSSA sample did not contain beads, it is plausible that there is some bead contamination in the system, giving rise to the bead signal in the MSSA sample.

By plotting the number of points appearing at the different phase angles in Figure 5B, we can see that *E. coli* and MSSA indeed present two distinct populations at 2 and 3 rad, respectively. Fitting the data using normal distributions we find that the mean phase value for the *E. coli* is 2.22 rad with a standard deviation of 0.55 rad (n = 325), while the mean phase value for MSSA is 3.09 rad with a standard deviation of 0.26 rad (n = 241). The two means are statistically significantly different as the *p*-value is of the order of 10^−90^. However, we note that the tails for the two distributions overlap at approximately the mean +1× standard deviation for *E. coli* and the mean −1× standard deviation for MSSA. Assuming a Gaussian distribution for the two populations, this means that we can correctly identify and categorize 84% of the bacteria in the sample.

Whether or not the differentiation method can be generalized to a larger number of different bacteria is still under investigation. Theoretically, different bacteria types have different membrane and cytoplasm structures and should therefore have different dielectric properties, though admittedly the differences are small. However, increasing the system sensitivity can be achieved by changing the channel geometry (e.g., in [30]), using a different set of frequencies or combining EIS with other dielectric based methods, such as DEP.

### 3.4. General Discussion

In the above we have presented how our device can detect and differentiate *E. coli* and *S. aureus* in water samples, however, the limit of detection (LOD) is far from what is required for such a sensor. The acceptable limit is 1 coliform per 100 mL water which is much lower than the ca. 500 bacteria/mL that our device can measure. Although *E. coli* and *S. aureus* were used as test bacteria in order to validate our method against the standard plate count, the purpose of this device is not to reach this type of sensitivity for a single bacteria species but to provide the total bacteria count in a water sample and detect changes to this number. Indeed, there are four main indicators used for water quality [31]: (a) Heterotrophic plate count (HPC), which refers to the number of culturable bacteria in a water sample, (b) Total coliform, (c) Fecal coliform and (d) *E. coli*. The presented sensor aims to address the first indicator, HPC [32]. Significant changes in HPC serve as an alert for possible deterioration of water quality, triggering further investigation. However, HPC only measures culturable bacteria, with the total number of bacteria being 1000 to 10,000 times higher [33]. The presented device can measure all bacteria in water, culturable or not, and therefore provides a fast and accurate measurement of the total bacteria count, that does not involve time-consuming culturing steps. The Danish limits for HPC are 5 bacteria per mL at 37 °C, which means a total of 5000 to 50,000 bacteria per mL, which is easily achievable with the presented device.

The maximum continuous and flawless operation time for this device installed in a water pipe was 14 days, which is not adequate for a real life application. Future work will focus on improving the stability of the system and on establishing the functionality in real-world samples.

## 4. Conclusions

In conclusion, a microfluidic chip was fabricated and used to distinguish *E. coli* from solid particles represented by polystyrene beads in both tap water as well as a diluted saline solution using multi-frequency impedance spectroscopy. Using the phase angle response at a frequency of 7 or 8 MHz, *E. coli* bacteria could be separated from polystyrene beads on a single transition basis. Moreover, we were able to distinguish between gram-positive and gram-negative bacteria using this method. Together with the system’s ability to detect bacteria concentrations with an LOD of 522 mL^−1^ and its ability to accurately determine a change in bacteria concentration, the system has been demonstrated to be an interesting candidate for further operational testing. All measurements were obtained with the label free technique of electrical impedance flow cytometry, and the results show great promise for using electrical impedance flow cytometry as the detection principle of the next generation of online and real-time water quality control sensors.

## Figures and Tables

**Table 1:** Details of the concentration determination experiments, along with the measured transitions and the calculated concentrations by EIS and plating.

Sample	Measurement Time (s)	Beads (#)	Beads (/mL)	*E. coli* (#)	*E. coli* (/mL)	Plate Count (/mL)
A	1551.46	377	191,202	7	6221	3000
B	1554.65	426	201,612	92	48,181	154,000
C	1551.71	398	192,603	224	118,006	326,000
D	1551.9	366	185,596	483	274,769	643,000
E	1861.85	445	186,478	843	396,767	1.00 × 10^6^

## Supplementary Materials

The following are available online at http://www.mdpi.com/1424-8220/18/10/3496/s1.
