# Supplementary material for: Bacteria Detection and Differentiation Using Impedance Flow Cytometry
Source: Sensors (Basel). 2018 Oct 17;18(10):3496. doi: 10.3390/s18103496 (PMC6210208; doi:10.3390/s18103496)
Supplement: Supplementary file 1 [file sensors-18-03496-s001.pdf]

Supplementary Data

Bacteria Detection and Differentiation using Impedance Flow Cytometry

Casper Hyttel Clausen [1], Maria Dimaki [2*], Christian Vinther Bertelsen [1,2], Gustav Erik Skands [2], Romen Rodriguez-Trujillo [3,4], Joachim Dahl Thomsen [2] and Winnie E. Svendsen [2]

S.1 Frequency selection

In order to select the low and high frequency used for the experiments we recorded the phase of the signal in the presence of different samples (beads, E.coli, S. aureus and L. Anisa) at different frequencies starting from 231 kHz to 10 MHz. No difference in the phase is observed at 231 kHz, as the membrane acts as an insulator and we only get information on the bacteria size and shape, which is similar for all bacteria. However, the phase can be differentiated at frequencies above 6 MHz. We have chosen 7 MHz as the high frequency signal, since we could also see that the preamplifier influences the measurements at higher frequencies.

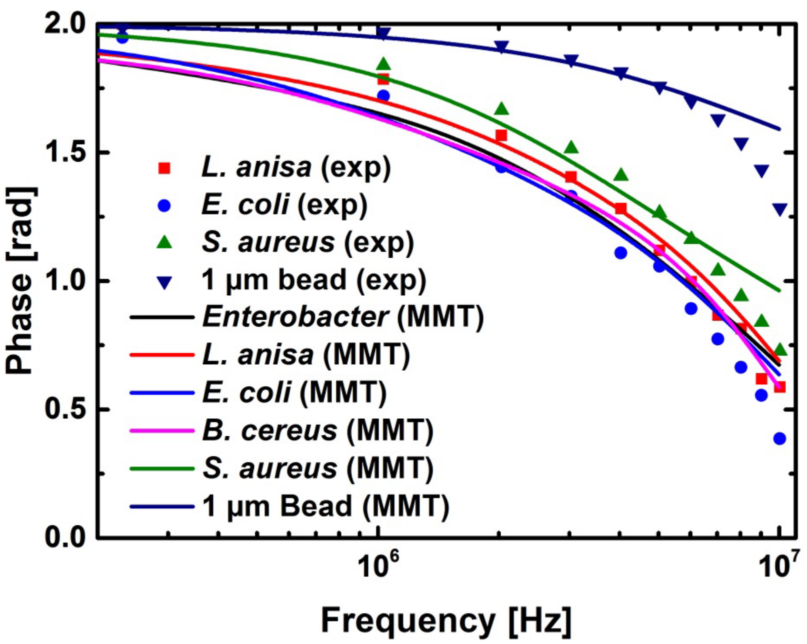

Figure S1. Theoretical (solid lines) and experimental (markers) data for a number of different bacteria in the frequency range 200 kHz to 10 MHz. Theoretical curves have been obtained using the Maxwell Mixture Theory (MMT).

S.2 Effect of solution ionic strength on the phase signal

All our experiments are conducted at a solution conductivity of 0.085 S/m, which is the average conductivity of drinking water in Denmark. However, the conductivity of drinking water can vary locally. In order to confirm that the phase signal from the bacteria remains the same despite this variation, we have conducted the measurements using E.coli in the conductivity range of 44-159 mS/m, which covers the range of conductivities found in drinking water. As shown in figure S2 the phase signal remains stable in this range of conductivities.

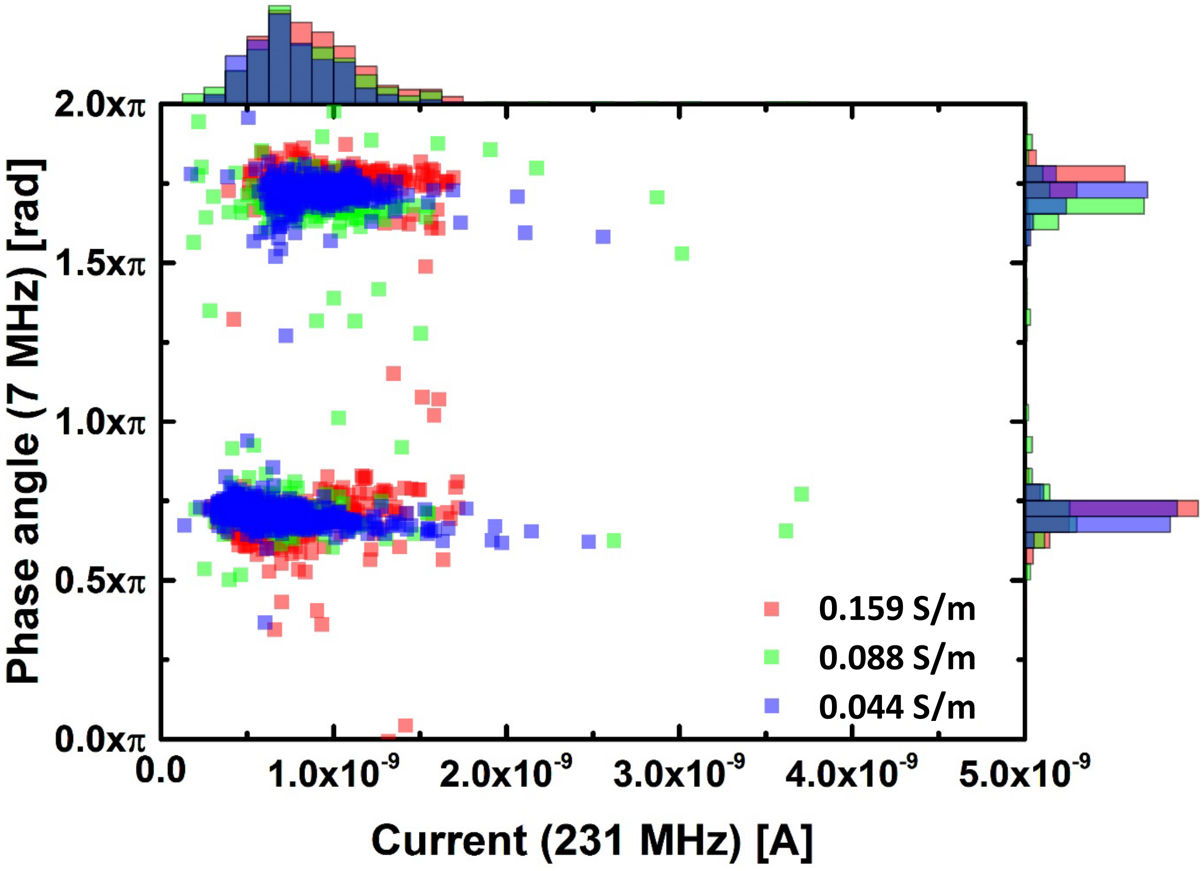

Figure S2. Phase shift at 7 MHz plotted against the low frequency signal of E. coli and 1 µm polystyrene beads in solutions with varying conductivities of 0.048 S/m, 0.088 S/m, and 0.159 S/m. The signal for E.coli appears between 0.5π and 1π.

If the solution conductivity increases further, e.g. by using PBS, then the phase signal is greatly affected, so that it is no longer possible to distinguish between bacteria and polystyrene beads (figure S3). This is, however, an extreme case, which is not found in drinking water.

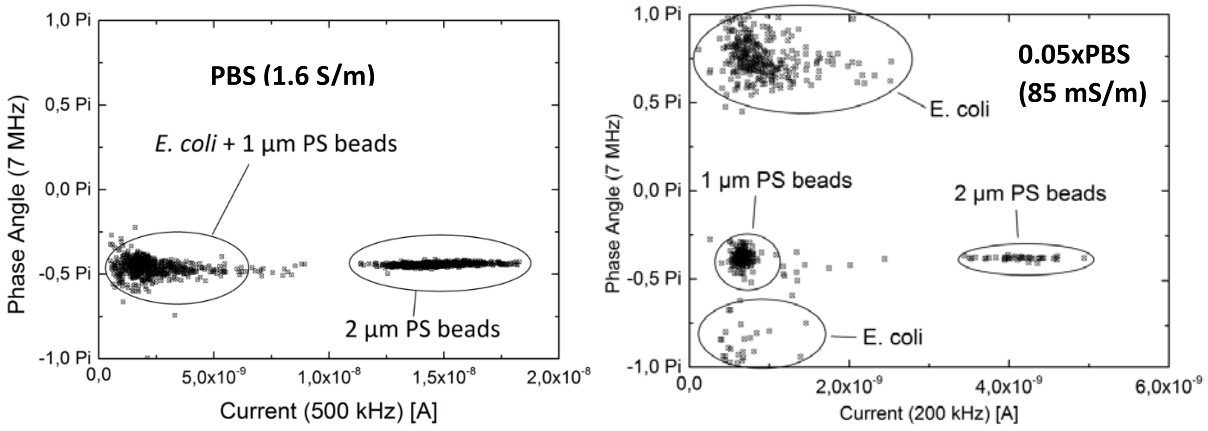

Figure S3. The phase angle at 7 MHz in PBS (left) and 0.05xPBS (right). It is not possible to distinguish the E.coli from polystyrene beads when the ionic strength of the solution is high. However, this is not a problem in drinking water, as the conductivity is never going to be that high.
